# The influence of high exercise levels on the coronary atherosclerosis profile by computed tomography angiography and outcomes

**DOI:** 10.1016/j.ajpc.2025.101044

**Published:** 2025-06-14

**Authors:** Gudrun M Feuchtner, Elias Ruf, Fabian Barbieri, Thomas Senoner, Johannes Deeg, Yannick Scharll, Gerlig Widmann, Pietro G. Lacaita

**Affiliations:** a Department of Radiology, Innsbruck Medical University, Innsbruck, Austria; b Deutsches Herzzentrum der Charité, Department of Cardiology, Angiology and Intensive Care Medicine, Hindenburgdamm 30, 12203 Berlin; c Department of Anästhesiology and Critical Care Medicine, Medical University Innsbruck, Austria

**Keywords:** Computed tomography, Exercise, Cardiovascular prevention, Risk factors, Atherosclerosis, Coronary artery disease

## Abstract

**Background:**

High exercise volumes may have deleterious effects on the cardiovascular system, and the upper thresholds for "safe" volumes are unclear.

**Objective:**

To evaluate if high-exercise volumes influence the coronary artery disease (CAD) profile by coronary CTA, cardiovascular outcomes, compared with traditional CVRF.

**Methods:**

802 patients (age 57.6 ± 10.95 years;38.1 % women) undergoing coronary CTA were enrolled. Self-reported exercise habits were collected: Type, volume (frequency/week and time/per unit); and years of training. Endpoints were: CTA: coronary stenosis severity (CADRADS); high-risk-plaque (HRP); coronary artery calcium score (CAC), and MACE.

**Results:**

478 subjects were included. 100 with high-(H)-exercise level (>3–5x/week and 1–3 h per unit) were propensity-score matched with 124 sedentary controls. The CVRF dyslipidemia (*p* = 0.393) and age were similar in both groups (*p* = 0.328), while arterial hypertension (*p* = 0.016), diabetes (*p* = 0.032), and the BMI (*p* < 0.001) were lower in athletes. CAC (80.5 vs 107.7 AU: *p* = 0.820, CADRADS: *p* = 0.394) and MACE-rates were not different. Follow-up was 3.95±1.0 years. After matching for sex, HRP was 1.6-fold less frequent in the H-group (17 % vs 32.8 %; *p* = 0.231; OR 1.58 (95 % CI: 0.787–3.222) and smoking was more prevalent in controls. There was no difference in HRP after adjusting for all CVRF. MET was mean 8.78 ± 3.5; weekly training volume 9 h. The majority (78 %) reported >5 -10 years of training.

**Conclusion:**

High-exercise levels (up to 9h/week) are safe - without a deleterious impact on the coronary atherosclerosis profile.

**Key findings/highlights**

Our data support the ACC/AHA and ACC guidelines for primary cardiovascular prevention through exercise recommendations, reinforcing their safety by demonstrating no deleterious effects - even at high exercise volumes averaging **9 h per week** – and showing no adverse outcomes.

The beneficial effects of exercise are modulated over a decrease in the major cardiovascular risk factors (CVRF) diabetes, hypertension, and body mass index (BMI)

Alt-text: Unlabelled box

**Figure:**
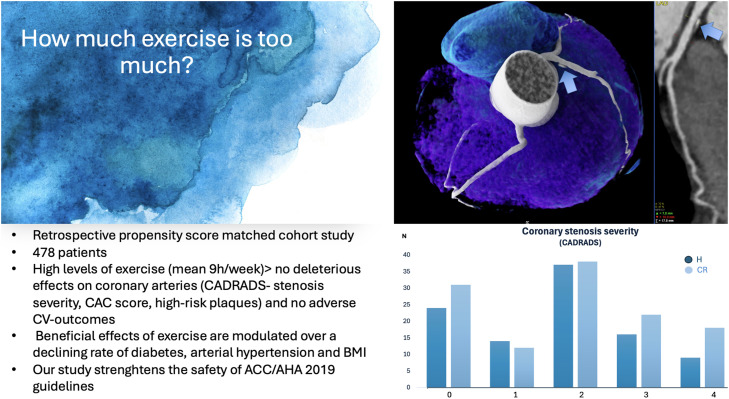
The influnece of high exercise levels on coronary atherosclerosis by coronary computed tomography angiography.

## 1 Introduction

Physical activity is recommended for cardiovascular prevention [1] due to its beneficial effects on cardiovascular outcomes. The ACC/AHA 2019 guidelines recommend a total volume of 150 min per week of moderate intensity or 75 min of vigorous exercise for cardiovascular prevention in healthy adults [1].

However, the MASTER@HEART study showed a higher non-calcifying mixed plaque burden in lifelong elite endurance athletes, raising concerns about deleterious effects in lifelong master endurance athletes with high training volumes [2]. Higher coronary artery calcium (CAC) scores were reported in lifelong athletes [3] and higher plaque volumes were observed in a study of 50 marathon runners [4]. In contrast, the MARC-2 study, which included 284 participants, reported a more benign plaque profile – characterized by less mixed plaques and more calcified plaques - on CTA, even among those with high training volumes exceeding 2000 metabolic equivalent of task (MET)-minutes/week [5]. Similar findings were observed in another cohort study [6] that recruited recreational (non-professional) endurance athletes with moderate-to-high training volumes.

Longitudinal data on cardiovascular (CV) outcomes, including major cardiovascular events (MACE), in athletes with high exercise volumes are lacking. Additionally, the relationship between the coronary artery disease profile assessed by CTA, CV outcomes, and endurance sport levels is not well studied [[4], [5], [6]]. Most existing studies have focused on the CAC score [7], with limited research using CTA.

Marathon running and other endurance sports, such as triathlon and cycling, fueled by social media trends, have led to a surge in non-professional endurance participants, many of whom reach high training volumes, comparable to elite athletes. The COVID-19 pandemic further accelerated the popularity of outdoor sports. For instance, participation in the Berlin Marathon increased from 36.544 to 40.050 over the past decade.

However, the thresholds of exercise volume and duration for obtaining beneficial vs. adverse effects on the cardiovascular system remain unclear [5,6]. Exercise resulted in a 44 % reduction of mortality in the Copenhagen Study [8] and showed an U-shaped relationship. The lowest mortality rates were observed at low levels of exercise (1–2.5 h of jogging/week at slow to moderate pace) [8], while higher training volumes had less favorable but similar outcomes than sedentarism. Although a dose-response relationship between the effect of exercise intensity and volume on the cardiovascular system has been reported [9], the exact threshold of deleterious effects is unknown.

Therefore, the purpose of this study was to evaluate whether high levels of endurance influence the coronary artery disease (CAD) profile quantified by coronary CTA and CV outcomes, compared to sedentary controls in an observational cohort study, and to assess the influence of the major CVRF. As secondary aim, we tested whether the effect of high exercise volumes on coronary arteries is direct or modulated through CV-risk factors.

## 2 Methods

### 2.1 Study cohort and population

Patients who underwent coronary CTA between 01/2010 and 10/2021 for clinical indications [10] were included in our retrospective observational cohort study. Institutional review board (IRB) approval for the database was obtained and written informed consent was waived. Symptomatic patients with unknown CAD referred to coronary CTA were included. Symptoms were defined as atypical, typical or non-specific chest pain, dyspnea, or prior testing (e.g., ECG treadmill stress test, echocardiography, or resting ECG) raising suspicion of obstructive CAD.

#### 2.1.1 **Inclusion criteria**

Patients were provided with a questionnaire about their exercise habits immediately prior to their CT exam, while in the waiting room. Only patients who complete the questionnaire were included. The questionnaire was approved by our local IRB, and first inquired whether any physical activity was performed (yes/no). The type of exercise was labelled as “E” = endurance (e.g. running, cycling, triathlon), “S” = strength (power and skill, such as ball sports only), and others (“O”: yoga, pilates, hula-hup or others. Exercise volume was stratified according by the frequency of training (*L* = low, 1 - 3x week, *M* = moderate, 3 - 5x week, and *H* = high, 5 - 7x week) and the time per unit (*L*= low, 20min-30 min, *M* = moderate: 1 h, *H* = high, 3 h or more). Regularity of training (yes/no) and the longitudinal time frame of training (years) was requested (less than 1 year, 1 - 3 years, 3 - 5 years, 5 – 10 years and more than 10 years), and whether they participated in competitions (yes/no). The weekly training volume was calculated for each group, and the MET-minutes/week. MET was defined as a measure of exercise volumes obtained by multiplying the MET score for the specific sport by the reported exercise volume (minutes/week) and calculated for the *E* = endurance group [11].

**1) High level group** (H) were defined as either reported regular training at frequency/time per unit levels M/H, M/M, H/M, and H/H were included, with a minimum of 3–5x/week and ≥1 h/session, i.e., ≥180 min of endurance training per week.
**2) Control group (CR):** All those who denied any physical activity were included and defined as “sedentary”. All others were stratified as “low” physical exercisers or other sports type (S, O) and were excluded from data analysis.

#### 2.1.2 Exclusion criteria

Prior coronary artery bypass grafting (CABG), percutaneous coronary intervention (PCI), acute coronary syndrome (ACS), or referrals for other clinical indications such as structural heart disease, congenital heart disease, or other interventional planning such as transcatheter aortic or mitral valve interventions.

#### 2.1.3 Computed tomography (CT)

**Coronary artery calcium (CAC) score:** A non-contrast ECG-gated CT scan with standardized parameters (detector collimation 2 × 64 × 0.6 mm; 120 kV; image reconstruction 3 mm slice width, increment 1.5), and prospective ECG-triggering in high-pitch dual-source mode was performed. The Agatston Score (AU) [12] of all coronary arteries was calculated with automated software (Cardiac CT, SyngoVIA, Siemens Healthineers).

**Coronary CTA** was performed with a 128-slice dual-source CT (Somatom Definition FLASH or DRIVE, Siemens) scanner: detector collimation 2 × 64 × 0.6 mm, z-flying spot, and rotation time 0.28 s). Prospective ECG-triggering was used in regular heart rates <65 bpm (70 % of RR-interval) and retrospective ECG-gating >65 bpm and irregular rates. An iodine contrast agent (Iopromide, Ultravist 370™) was injected intravenously (flow rate 4–6 ml/*s* + 40cc saline), triggered into the arterial phase using bolus tracking, into the ascending aorta. Contrast volume ranged from 65 −120cc and was adjusted to the patients’ weight using a fixed scheme. Axial images were reconstructed with 0.75 mm slice width (increment 0.4/medium-smooth kernel B26f) during best diastolic and systolic phase. Curved multiplanar reformations (cMPR) and oblique interactive MPR using client-server based 3-D post-processing software (CardiacCT, SyngoVia™, Siemens Healthineers) were generated and the following CTA-parameters measured:

1) **Coronary stenosis severity** was scored visually according to CAD-RADS™ [13] classification as minimal [1]<25 %, mild [2] 25 - 49.9 %, moderate [3] 50 - 69.9 %, severe [4] ≥70 % - 99 % and [5] occluded 100 % on a per-coronary segment-base (AHA-modified-17-segment classification) assisted by quantitative stenosis measurement using curved multiplanar reformations (cMPR). Obstructive disease was defined as >50 % stenosis (CADRADS 3 and 4).
2) **Coronary plaque phenotypes**: High-risk-plaque (HRP) analysis was performed:

Low attenuation plaque (LAP) was defined as a hypoattenuating lesion with <150 HU. CT-density was screened with the “pixel lens” [14] and the lowest HU were recorded [22]. LAP<30HU was defined as lipid-rich necrotic core [15], and LAP<60 HU as fibrofatty. Napkin-ring sign was defined as an outer high-density rim with an inner hypodense area [16]. Spotty calcification (SC) was defined as a calcification of less than 3 mm. Positive remodelling was defined as a remodelling index of >1.1. A patient was labelled as “HRP” if a minimum of 2 criteria was present, and if at least one LAP <30HU or LAP <60 HU was present per patient. Coronary CTA analysis was performed by one highly experienced reader (>5–10 years cardiac CT).

#### 2.1.4 Clinical data collections

Conventional cardiovascular risk factors (CVRF) were collected defined according to standardized European Society of Cardiology (ESC) criteria: arterial hypertension (systolic blood pressure>140 mmHg or diastolic>90 mmHg), dyslipidemia, positive family history (myocardial infarction or sudden cardiac death in an immediate male relative<55 years or female<65 years, smoker (active: current or quit less than 6 months before CCTA examination and former), and diabetes [[17], [18]–19]. Medication potential affecting coronary artery disease was collected (acetylsalicylicacid and statins).

#### 2.1.5 Statistical methods

Statistical analysis was performed using SPSS™ software (IBM, V25.0, SPSS Inc., Chicago, USA). A propensity score matchmaking model was utilized to reduce the possibility of selection bias and confounding. The derived propensity-score was then utilized for matchmaking by using a 1:1 nearest neighbor matchmaking process with a matching tolerance of 0.05.

Univariate binary logistic regression analysis was performed to test for the influence of the variable H (high-level of exercise) for the endpoint HRP – in stepwise models 1) without matchmaking 2) with matchmaking for a) sex b) non-modifiable CVRF gender and positive family history and c) all CVRF (age, body mass index, and the major cardiovascular risk factors (smoking, arterial hypertension, positive family history, dyslipidemia and diabetes). Second, the binary logistic regression analysis was repeated to test for associations between CAC (tested separately for the thresholds of >0 and >100 AU) and high exercise volumes. Chi-square test was performed to assess differences between the 4 CAC categories (0, 1–99, 100–299, >300) and the groups H and sedentary controls.

Quantitative variables are expressed as means ± standard deviation (SD) or as median (Interquartile Range, IQR), and categorical variables as absolute values and percentages. The normal distribution of data was tested. The Chi-square was applied for differences in categorical data between the 2 groups, and the Fisher´s exact test, in the sample size in one field was less than 5. The independent *t*-test for normally distributed data and the Mann-Whitney-U for non-normally distributed data (CAC, CADRADS).

## 3 Results

Of 802 patients (age 57.6 ± 10.95 years, 38.1 % females) enrolled in our database, 478 completed the exercise habits questionnaire. A total of 100 individuals with high (H) exercise levels (defined as more than 3–5x/week with 1–3 h duration per unit) were compared to 124 sedentary controls. 253 subjects with a low level of exercise and strength sport type or other sport types, were excluded. Table 1 shows the profile of the finally included study cohort (*n* = 224). In the H-Group, the average weekly volume of training was 9 h per week. (3–5 x/week, 1–3 hour/session = 3–15 h/week: average 9 h/week). MET was mean 8.78 ± 3.5.

**Table 1:** Study population (*n* = 224).

**Age** (years)	57.5 ± 10.8
**Females**	89 (39.7 %)
**Body mass index** (kg/m^2^)	26.5 ± 4.6
**Smoking**	57 (25.4 %)
**Arterial hypertension**	104 (46.4 %)
**Pos. Family History**	108 (48.2 %)
**Dyslipidaemia**	115 (51.3 %)
**Diabetes**	22 (9.8 %)
**Medication**	
Statins	66 (29.4 %)
**Lipid panels**	
Cholesterol (mg/dl)	205.1 ± 48.7
LDL-c (mg/dl)	128.7 ± 43.0
HDL (mg/dl)	58.6 ± 19.4
TG (mg/dl)	132.3 ± 88.9

Abb. LDL-*c* = low density lipoprotein, HDL = high density lipoprotein, TG = triglycerids, ASA= acetylsalicylacid. RR = Blood pressure (arterial). Sys = systolic. Dia = diastolic. Categorical data are shown as N ( %). *N* = counts.

There were 32 (32 %) runners (*n* = 2 marathons (*n* = 1 halfmarathon and *n* = 1 ultramarathon runner), 18 (18 %) cyclists (5 mountain bikers and 13 road cyclists), 24 (24 %) mountain endurance athletes (skimountaineering/cross-country skiing and mountain-biking), 2 (2 %) swimmers 4 mountain hikers or 2 nordic walking (6 %), and 18 (18 %) mixed endurance athletes (different types). Exercise duration: A total of 69 (69 %) performed endurance sport for more than 10 years, 9 (9 %) for 5–10 years, and 7 (7 %) between 1–5 years and 4 (4 %) less than 1 year, and 11 (11 %) did not report the duration of training. Competitive level: 5/100 (5 %) in the high-exercise group reported regular participation in competitions, 75/100 (75 %) defined themselves as “leisure-time” athletes, and 20/100 (20 %) did not report whether or not they participated in competitions.

Table 2 shows the CVRF and CTA results for the groups with high (H) exercise vs sedentary controls. The prevalence of arterial hypertension (37 % vs 54 %, *p* = 0.016), diabetes (5 % vs 13.7 %, *p* = 0.032), and the BMI (24.3 ± 3.21 vs 28.2 ± 4.75, *p* < 0.001) were significantly lower in endurance athletes compared to sedentary controls, while CAC (80.5 vs 107.7 AU, *p* = 0.820) and coronary stenosis severity (CADRADS, *p* = 0.394) (Fig. 1a, Fig. 1b) and all cardiovascular outcome endpoints (death, ACS, revascularization rate) were not different (Table 3). Mean follow-up time was 3.95 years ± 1.0 years (range, 1- 6). Fig. 2, Fig. 2b illustrates two case examples of patients and their CT scan findings.

**Table 2:** Comparison of high exercise levels with sedentary controls (CR): CVRF and the coronary artery disease profile by CTA: high-risk plaque (HRP), coronary stenosis severity (CADRADS and obstructive disease>50 % stenosis rate), CAC score.

	High	CR	p-value
	*N* = 100	*N* = 124	
**Age** (yrs)	56.7 ± 10.9	58.1 ± 10.6	0.328
**Females**	29 (29 %)	60 (48.4 %)	0.008
**BMI** kg/m^2^	24.3 ± 3.2	28.2 ± 4.7	<0.001
**Cardiovascular risk factors (CVRF)**			
**Nicotine**	17 (17 %)	40 (32.3 %)	0.012
**Arterial hypertension**	37(37 %)	67 (54.0 %)	0.016
**Pos Family History**	38 (38 %)	70 (56.5 %)	0.006
**Dyslipidemia**	48 (48 %)	67 (54.0 %)	0.393
**Diabetes**	5 (5 %)	17 (13.7 %)	0.032
**CTA endpoints**			
**HRP**	17 (17 %)	26 (20.9 %)	0.435 OR: 1.294 (95 % CI 0.657–2.59)
**CAC (AU)**	Mean 80.5 Median 5.6 (IQR 61)	Mean 107.7 Median 4.55 (IQR 86)	0.820
**CADRADS**	Median 2 (IQR 2)	Median 2 (IQR 3)	0.394
**0**	24	31	
**1**	14	12	
**2**	37	38	
**3**	16	22	
**4**	9	17	
**5**	0	1	
**Obstructive CAD>50 % stenosis**	25 (25 %)	40 (32.3 %)	0.235 (OR: 1.426, 95 % CI 0.790–2.595)

Abb.: HRP = High Risk Plaques. *N* = counts. BMI = Body Mass Index. AU = Agatston Units. OR = Odds Radio. CAD = coronary artery disease. CADRADS = coronary artery disease reporting system for coronary artery stenosis severity. CAC = coronary artery calcium score. AU = Agatston Units.

**Fig. 1a:**
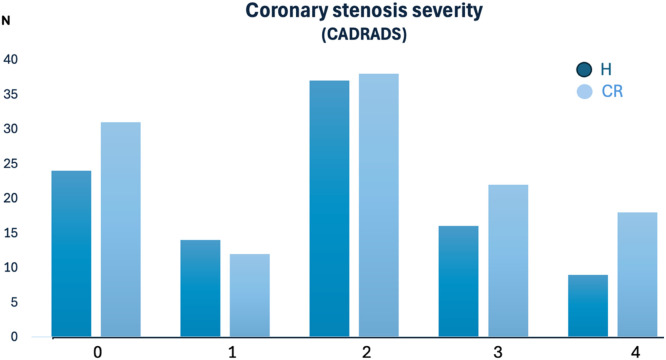
**Coronary stenosis severity (CADRADS):** There was no difference between participants with a high (H) exercise levels compared to sedentary controls (CR) (*p* = 0.394). CADRADS 0 = no CAD, 1 = minimal coronary stenosis 1–24 %, 2 = mild stenosis 25–49 %, 3 = moderate, 50–69 %, group 4 = severe stenosis 70–99 % and 100 % occlusions (CADRADS 4 + 5). Y-axis: counts (N).

**Fig. 1b:**
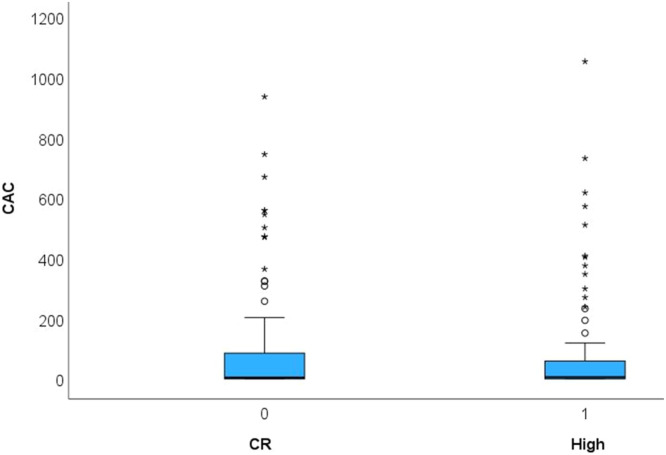
The **coronary artery calcium score (CAC)** was not different between subjects with a high exercise volume and sedentary controls (CR) (*p* = 0.820). CAC is displayed in Agatston Units (AU).

**Table 3:** **Clinical outcomes in the high-exercise (High) volume group vs. sedentary controls: major cardiovascular events (MACE)** Death, ACS (STEMI/NSTEMI), and ICA >50 % (+ revascularization rate: PCI or CABG).

	High	CR	p-value
	*N* = 100	*N* = 124	
**Death**	0 (0 %)	1 (0.8 %)	>0.999
**ACS (STEMI or NSTEMI)**	2 (2 %)	3 (2.4 %)	>0.999
**ICA > 50 % stenosis**	8 (8 %)	13 (10.5 %)	0.686 HR 1.345 (0.534–3.552)
**Revascularization rate (PCI or CABG)**	7 (7 %)	13 (10.5 %)	0.501 HR 1.55 (0.598–4.307)
PCI	6 (6 %)	11 (8.9 %)	
CABG	1 (1 %)	2 (1.6 %)	
conservative	1 (1 %)	0 (0 %)	
**ICA yes**	22 (22 %)	27 (21.8 %)	0.095

Abbreviations: ICA = invasive coronary angiography. ACS = acute coronary syndrome. STEMI = ST-elevation myocardial infarct. NSTEMI = non-ST-elevation myocardial infarct. PCI = percutaneous coronary intervention. CABG = coronary artery bypass grafting. HR = hazard ratio (95 % Confidence intervals).

**Fig. 2:**
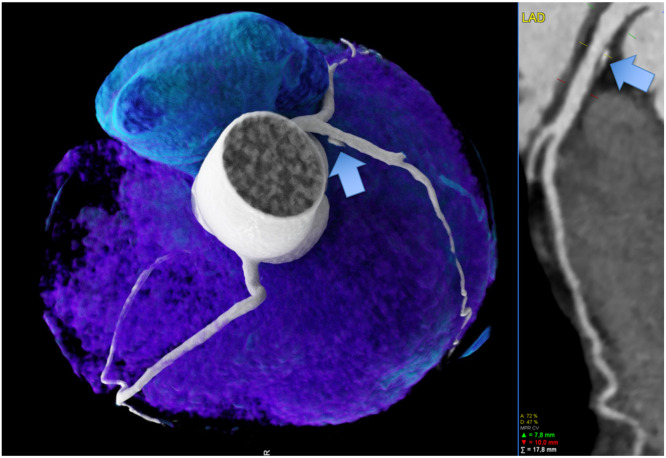
59 years-old-male cyclist with a high-level of training over more than 10 years, no chest pain but referral to coronary CTA due to polytopic ectopic ventricular extrasystole during exercise. CTA showed a predominantly calcified plaque in the proximal LAD (arrows) with less than 50 % stenosis (CADRADS 2). 1 CVRF: arterial hypertension. Left: 3D volume rendering technique (VRT) and right: curved multiplanar reformation (cMRP) showing the left anterior descending (LAD) coronary artery.

**Fig. 2b:**
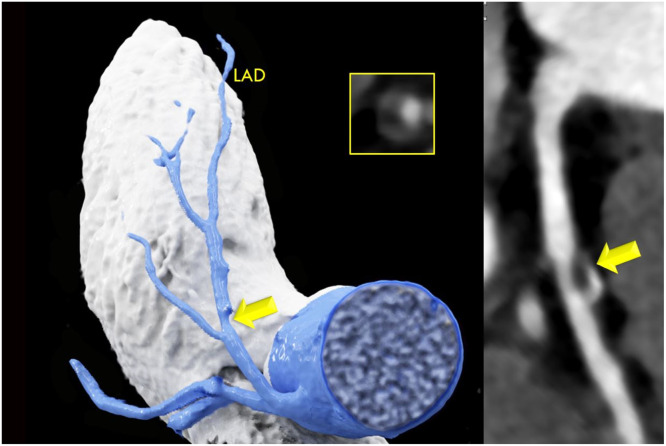
41-year-old sedentary male with 2 CVRF (smoking and dyslipidemia), and a high-risk plaque (HRP) in the proximal LAD (yellow arrow). Low attenuation plaque (LAP) less than 30 HU, positive remodelling, Napkin-Ring sign and 46 % diameter stenosis (CADRADS 2). Left: curved multiplanar reformation (cMPR) and right: 3D Volume rendering technique (VRT) spyder view.

High-risk plaque (HRP) prevalence was slightly lower (17 % vs. 20.9 %, *p* = 0.435, OR: 1.294; 95 % CI: 0.657–2.59) in high-level endurance athletes, and the obstructive disease rate (25 % vs. 32.3 %, *p* = 0.235, OR: 1.426, 95 %CI: 0.79–2.595) was lower. However, there were significantly more women (29 % vs. 48.4 %, *p* = 0.008) in sedentary controls and more smokers (17 % vs. 32.3 %, *p* = 0.012). The CVRF dyslipidemia and age were equal in both groups (H vs 0: 48 % vs. 54 %, *p* = 0.393)(age:56.7 vs. 58.1 years, *p* = 0.328). After balancing the groups for sex only, the prevalence of HRP was 1.58-fold lower in the high-exercise group (17 % vs. 32.8 %, *p* = 0.231; OR 1.582: 95 % CI: 0.787–3.222) with 29/99 (29.2 %) vs. 34/97 (34.3 %) (*p* = 0.447).

Propensity score matching was performed for 2 models (Table 4). First, the groups were matched for the non-modifiable CVRF sex and positive family history and smoking (Table 4a). After matching for sex, positive family history, and smoking, the trend for a slightly higher rate of HRP and obstructive disease ceased completely, while the CVRF arterial hypertension and diabetes were still higher (Table 4a). There was no difference in CAC and CADRADS. Second, the groups were matched for all CVRF (Table 4b). There was no difference in the CAD profile by CTA (CAC, CADRADS), and no difference in the HRP prevalence.

**Table 4a:** High level of endurance training: Results after propensity score matching for sex, smoking and positive family history: The CVRF arterial hypertension and diabetes (*p* = 0.048 and *p* = 0.017) and BMI (*p* < 0.001) were still higher, but the CAD profile by CTA (HRP, CAC and CADRADS) was not different. The trend towards less HRP and a lower obstructive disease rate completely ceased.

	High	CR	p-value
	*N* = 74	*N* = 74	
**Age** (yrs)	57.5 ± 10.9	57.5 ± 11.2	0.497
**Women**	28 (37.8 %)	27 (36.4 %)	>0.999
**BMI** (kg/m^2^)	24.3 ± 3.3	28.6 ± 5.3	<0.001*
**Cardiovascular risk factors (CVRF)**			
**Smoking**	17 (22.9 %)	21 (28.4 %)	0.573
**Arterial hypertension**	29 (39.2 %)	41 (55.4 %)	0.048*
**Pos. FH**	38 (51.4 %)	35 (47.3 %)	0.743
**Dyslipidemia**	35 (47.3 %)	38 (51.4 %)	0.743
**Diabetes**	3 (4 %)	13 (17.6 %)	0.017*
**CTA endpoints (CAD profile)**			
**HRP**	14 (18.9 %)	15 (20.3 %)	>0.999
**CAC (AU)**	Mean 74.6 ± 180 Median 6.35 (IQR 47)	Mean 88.6 ± 172 Median 4.55 (IQR 85)	0.705
**CADRADS**	Median 2 (IQR 1)	Median 2 (IQR 2)	0.147 (Mann Whitney)
**0**	16	14	
**1**	10	7	
**2**	30	26	
**3**	11	14	
**4**	7	13	
**5**	0	0	
**Obstructive CAD>50 % stenosis**	18 (24.3 %)	27 (36.5 %)	0.153 (OR: 0.56; 95 %CI: 0.272–1.144)

Abb.: HRP = High Risk Plaques, *N* = counts. BMI = Body Mass Index, OR = Odds Ratio, CAD = coronary artery disease, yrs = years, CVRF = cardiovascular risk factors, CTA = computed tomography angiography. CAC = coronary artery calcium score. AU = Agatston Units. CAD = coronary artery disease. IQR = Interquartile Range. Pos. FH = Positive Family History.

**Table 4b:** **Results after propensity score matching for all CVRF (gender, smoking and positive FH, dyslipidemia, diabetes):** There was no difference in the CAD profile by CTA (HRP, CAC and CADRADS) between those with high exercise levels vs controls (CR).

	High	CR	p-value
	*N* = 70	*N* = 70	
**Age** (yrs)	57.7 ± 11.1	58.2 ± 11.7	0.797
**Women**	27 (38.6 %)	25 (35.7 %)	0.726
**BMI** (kg/m^2^)	24.3 ± 3.4	28.3 ± 4.9	<0.001*
**CVRF**			
**Smoking**	17 (24.2 %)	18 (25.7 %)	0.845
**Arterial hypertension**	32 (45.7 %)	34 (48.6 %)	0.735
**Pos. FH**	36 (51.4 %)	32 (45.7 %)	0.499
**Dyslipidemia**	38 (54.2 %)	34 (48.6 %)	0.499
**Diabetes**	5 (7.1 %)	8 (11.4 %)	0.382
**CTA endpoints (CAD profile)**			
**HRP**	13 (18.6 %)	14 (20 %)	>0.999 (OR: 0.913 (0.387–2.141)
**CAC (AU)**	Mean 88.7 ± 189 Median 5.5 (IQR 76)	Mean 86.7 ± 170 Median 4.1 (IQR 85)	0.891
**CADRADS**	Median 2 (IQR 1)	Median 2 (IQR 2)	0.272 (Mann Whitney) 0.269 (Kruskal Walllis)
**0**	17	15	
**1**	10	5	
**2**	24	26	
**3**	12	12	
**4**	7	11	
**5**	0	0	
**Obstructive CAD>50 % stenosis**	19 (27.1 %)	23 (32.8 %)	0.580 (OR: 0.76; 95 %CI: 0.365–1.852)

**Logistic regression models:** Binary logistic regression analysis showed no influence of “H” exercise levels on the feature “HRP” for all models a) the entire cohort, and b) after matching for non-modifiable CVRF sex and positive family and c) after propensity score matching for gender, smoking and all other major CVRF (OR 1.167, 95 % CI: 0.430 – 3.169, *p* = 0.762).

Second, the logistic regression models outlined above were repeated in the same fashion for the variable “CAC” (instead of HRP). No association were observed for all models a) b) and c) after propensity score matching for gender, smoking and all other major CVRF, as follows: For a threshold of CAC >0 AU, OR was 0.909 (95 % CI: 0.461 - 1.791, *p* = 782), and for a threshold of >100 AU, OR was 0.828 (95 % CI: 0.369 – 1.861, *p* = 0.648). CAC was categorized into 4 categories (0, 1–99, 100–299, ≥300 AU). There was no difference between the 4 CAC categories and the groups H and sedentary controls (Chi-Square, *p* = 0.648).

## 4 Discussion

First, our study showed no deleterious effects of high levels of exercise, averaging 9 h of training per week, on the coronary artery disease profile (CAC, coronary stenosis severity ((CADRADS)) and high-risk plaque) assessed by CTA. Second, no adverse CV outcomes were observed at long-term F/U interval of approximately 4 years. The training volume in our cohort exceeds the minimal exercise ACC/AHA recommendations (>180 min /week) for primary prevention [1]. However, the lifetime accumulated exercise volumes in our cohort of recreational athletes were presumably lower than those of professional elite master athletes with lifelong high training volumes, as described in other cohorts by De Bosscher [2] and Merghangi [3] et al.

Traditional CVRFs, particularly arterial hypertension and diabetes were the main modifiers for the reduced CV risk induced by exercise. Exercise reduces arterial hypertension by improving endothelial function, decreasing arterial stiffness, and modulating autonomic balance [20]. Similarly, regular physical activity enhances insulin sensitivity and glycemic control, which in turn mitigates the pro-inflammatory and atherogenic effects of diabetes [21]. These benefits were reflected in lower rates of arterial hypertension, diabetes, and BMI, as well as reduced smoking in individuals performing moderate-to-high endurance sport on a non-professional level in our cohort and resulted in a 1.6-fold reduction in the rate of HRP and a 1.2-fold decrease in obstructive disease. However, this trend was confounded by a higher proportion of smokers in the sedentary group.

Sex is another important factor influencing the features of coronary atherosclerosis. Due to the protective effects of estrogen in females, the onset of atherosclerosis occurs approximately 12 years later as compared to males [22], leading to sex-specific differences in plaque composition (non-calcified vs. calcified) at the same age. Despite women having lower total plaque burden compared to males, their outcomes are worse [23]. Women are more frequently underdiagnosed for CAD, due to a higher rate of atypical symptoms, a higher rate of nonobstructive disease, and microvascular dysfunction [23].

The risk of HRP decreased 1.6-fold after matching for gender, while the other CVRF including smoking [24] and diabetes, remained higher. These CVRFs have a major influence on the atherosclerosis profile; especially smoking [24] is associated with a higher prevalence of HRP. The rate of dyslipidemia and age were matched in our cohort.

After adjusting for all cardiovascular risk factors (CVRF) in athletes with a high level of exercise, the coronary plaque profile was similar to that observed in the MARC-2 study. In the MARC-2 study, exercise volumes of 2000 MET-min per week did not lead to adverse effects on the coronary artery disease profile [5].

Further, we observed that CAC scores were not different between both groups, and there was no influence of the variable “sport” on logistic regression models consistent with literature: In the Cooper Study [25], no impact of different recreational exercise levels on CAC progression, even at higher volumes, was found. However, higher CAC scores have been reported in lifelong master elite athletes [3] and professional marathon runners [4], indicating that extremely high training volumes over the entire lifespan may have adverse effects. Nonetheless, the exact upper threshold of exercise, in which coronary calcification increases, remains unclear. The coronary artery calcium score is a valuable prognosticator of adverse cardiovascular outcomes and is recommended for CV risk stratification [26]. However, recent data have revealed that calcified plaque, with an increasing plaque density up to 1k HU [27], results in lower ACS event rates as compared to non-calcified and mixed coronary plaque burden, while the presence of very low-density LAP <30HU, quantified by coronary CTA, is an independent prognosticator for higher CV risk [15].

Importantly, in contrast to prior studies [6], we performed a long-term follow-up of approximately 4 years and tested for the influence of CVRF by using the most sensitive method, propensity score matching. Our data therefore strengthen the safety of high exercise levels at a leisure time level (9 h/week) in terms of not causing more CV events. However, the low MACE rates must be acknowledged as limitation for the interpretation of long-term follow-up data.

The majority of participants had been practicing sports for over 10 years (69 %). However, we did not distinguish between lifelong (30 years) vs. late-onset athletes, such as in the Master@HEART study [2], which compared these two groups. That study found adverse plaque profiles, specifically a higher non-calcified plaque burden in lifelong elite athletes, even after adjusting for all CVRF, pointing at potential harmful effect of very high training volumes on coronary vessel walls from over an extended period at a professional level.

The majority of athletes in our cohort described themselves as “recreational”, and only a minority reported themselves as “competitive”. In general, leisure time athletes do not reach as high training volumes as professional athletes, despite some individuals may reach similar levels. There were only a few subjects (*n* = 2) who reported having participated in a half – and an ultramarathon. Male marathon runners (*n* = 50) had higher non-calcified plaque, calcified plaque, and total plaque volume in a prior study [4], while diameter stenosis was not significantly different, similar to our study, in which stenosis severity was not different compared to sedentary in several models, with and without matching for CVRF.

Finally, exercise intensity and volumes may have a different impact on CAC. Higher intensity has recently been shown to be associated with lower CAC, and longer durations increase CAC [28]. Similarly, the MARC-2 study found that exercise intensity but not volume was associated with the progression of coronary atherosclerosis during 6 years of follow-up. However, interestingly, very vigorous intensity exercise (>9 MET/week) was associated with greater CAC and calcified plaque progression, whereas vigorous intensity exercise was associated with less CAC progression (6–9 MET/week) [29] - beeing consistent with the training volume of our study cohort.

In contrast to the MARC-2 [29] and the MASTER@HEART study [2], which enrolled asymptomatic athletes, our population is distinct: We recruited symptomatic patients with low-intermediate pre-test probability of CAD who were referred to coronary CTA for clinical indications (either chest pain or other abnormal pre-test findings on treadmill). However, this cohort is most representative of endurance athletes at risk for sudden cardiac death (SCD) and in whom screening for occult CAD is highly important to prevent fatal events. SCD during exercise, in particular during competitive sports events at maximal exertion, is most commonly caused by occult CAD (in 80 % of males >35 years) with higher incidence rates in males compared to females and a relative risk ranging from 9:1 to 3:1 [30]. Therefore, coronary CTA is recommended for screening of underlying CAD in males over 35 years according to ESC 2020 guidelines [30].

Our cohort comprised different endurance sport types - cycling, running, or both - and is also distinct from other study cohortsdue to our alpine geographical location. Notably, one third of the population engaged in sports involving altitude elevation gain such as mountain biking or ski-mountaineering. Also, road cyclists commonly incorporate elevation gain into their routes. This terrain naturally leads to greater time spent in higher-intensity zones compared to flat running or cycling. In contrast, the proportion of participants involved in low-intensity endurance activities, like Nordic walking, was minimal (4 %).

The percentage of competitive athletes was low, therefore our cohort is not representative for professional competitive elite athletes. Long-term leisure time physical activity results in a reduction of all-cause and cardiovascular mortality [31]. However, very high long-term leisure-time physical activity (LTPA) (>3840 kcal/week), studied in 62 asymptomatic men over 25 years, was associated with elevated inflammatory biomarkers (hs-CRP, oxidized-LDL, IL-6) and increased intima-media thickness (IMT); while men with high LTPA (2050–3840 kcal/week) had lower CAC scores and lower levels of inflammation [32].

### 4.1 Limitations

This was a retrospective study with an inherent selection bias. Endurance exercise intensity (defined as % VO2 peak or % peak heart rate) [30] was not measured, however, subjects with low-intensity exercise such as walking and those who reported low levels or irregular exercise habits were excluded. Exercise habits were self-reported. MACE rates were low, limiting statistical power for long-term outcome analysis. The number of smokers and competitive athletes was relatively small.

## 6 Conclusion

High levels of exercise (mean 9 h per week) are associated with lower rates of diabetes, hypertension, and reduced BMI, but do not impact coronary stenosis severity, coronary artery calcium (CAC), or cardiovascular outcomes. Accordingly, our study confirms that even high volumes of non-professional leisure-time exercise have no harmful effects on coronary artery morphology or plaque characteristics.

## Clinical relevance

Our findings reinforce ACC/AHA and ESC guidelines [1,30] for minimal exercise volumes, demonstrating that even higher exercise volumes (up to 9 h/week) at a non-professional level remain safe and do not increase cardiovascular risk. This upper threshold may serve as a practical benchmark for prescribing exercise in clinical practice for primary prevention.

## Author statement

The submission is not under consideration elsewhere. No financial support was received. There is no conflict of interest. There are no disclosures for all authors.

## CRediT authorship contribution statement

**Gudrun M Feuchtner:** Writing – review & editing, Writing – original draft, Validation, Supervision, Software, Investigation, Data curation, Conceptualization. **Elias Ruf:** Writing – review & editing, Investigation, Data curation. **Fabian Barbieri:** Writing – review & editing, Validation, Data curation, Conceptualization. **Thomas Senoner:** Writing – review & editing, Data curation. **Johannes Deeg:** Writing – review & editing, Data curation. **Yannick Scharll:** Writing – review & editing, Data curation. **Gerlig Widmann:** Writing – review & editing, Supervision, Data curation. **Pietro G. Lacaita:** Writing – review & editing, Data curation.

## Declaration of competing interest

The authors declare that they have no known competing financial interests or personal relationships that could have appeared to influence the work reported in this paper.
